# Investigation of Electrode Cap Life Made of New Cu–Cr–Zr Copper Alloys with Scandium Addition Dedicated for Resistance Spot Welding of Galvanized Steel Sheets

**DOI:** 10.3390/ma18214950

**Published:** 2025-10-30

**Authors:** Krystian Franczak, Paweł Kwaśniewski, Grzegorz Kiesiewicz, Wojciech Ściężor, Michał Sadzikowski, Szymon Kordaszewski, Piotr Micek, Damian Kuca, Rafał Pestrak

**Affiliations:** 1 Faculty of Non-Ferrous Metals, AGH University of Krakow, 30-059 Kraków, Poland; 2 Faculty of Mechanical Engineering and Robotics, AGH University of Krakow, al. A. Mickiewicza 30, 30-059 Krakow, Poland; 3 KUCA Sp. z o.o., 73-110 Stargard, Poland

**Keywords:** Cu–Cr–Zr alloys, resistance welding process, diffusion layer, RSW electrode life, scandium

## Abstract

This study presents results on developing new copper alloys for electrode caps used in resistance spot welding (RSW) of galvanized steel sheets. Two copper alloys—CuCr0.7Zr0.05 and CuCr0.3Ni0.1Zr0.05—were modified with scandium (Sc) additions of 0.01 and 0.05 wt. %. Within this article, the influence of scandium content on Vickers hardness (HV) and electrical conductivity during alloy aging was investigated. In addition, the electrode life of the produced electrodes was subjected to detailed analysis. The results demonstrated that Sc modification enables an increase in hardness with only a minimal decrease in electrical conductivity. Moreover, Sc-modified electrodes exhibited a significantly reduced diffusion layer thickness in the electrode material, which led to lower degradation of the working face geometry and reduced material loss compared with commercial Cu–Cr–Zr electrodes. Mechanical testing showed that spot joints produced with the new electrodes exceed the minimum shear–tension strength requirements even after 500 welds. These results confirm that the proposed alloying approach extends electrode cap life and improves spot weld quality, supporting its application in industrial RSW.

## 1. Introduction

Resistance spot welding (RSW) of galvanized steels is widely used across machinery, aerospace, shipbuilding, home appliances, and the automotive industry. Among these, the automotive sector accounts for the largest share of RSW applications, primarily in galvanized steel body-in-white assembly. It is estimated that a single passenger car contains between 3000 and 6000 spot welds. Given around 80 million vehicles produced worldwide each year, the total number of spot welds is on the order of 400 billion [1,2,3,4].

**Figure 1:**
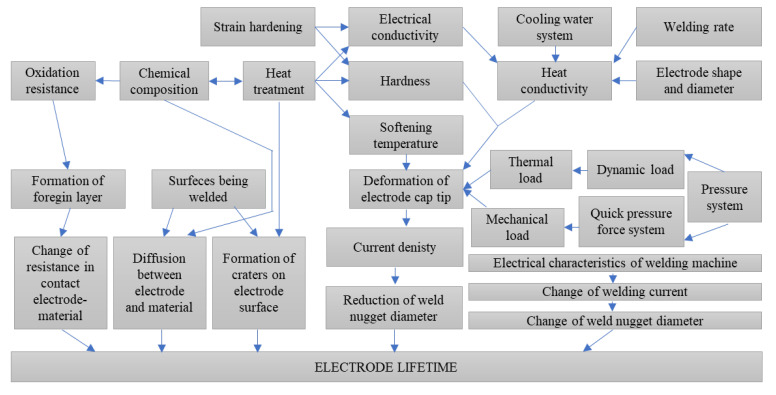
Factors influencing the service life of electrode caps in the resistance spot welding process [8].

Electrode cap life is a key factor influencing spot weld quality and directly affects production costs due to the downtime required for electrode replacement on production lines. The key factors determining electrode life include chemical composition, mechanical, electrical, and thermal properties, electrode shape and geometry, the condition of the working surfaces, and various welding process parameters, as illustrated in Figure 1 [5,6,7,8,9,10]. Depending on the welding parameters, the lifetime of electrodes during the welding of galvanized components is typically 300–500 welds without surface cleaning or current stepping. When such procedures are applied, electrode life can be extended to as many as 2500 welds by removing surface deformation through machining [11,12,13,14,15,16].

The most common and AWS-(American Welding Society)-recommended materials for electrode caps used in spot welding of galvanized sheets are Cu–Cr, Cu–Zr, and Cu–Cr–Zr alloys from group A2/2, as well as the Cu–Al_2_O_3_ composite material from group C20, owing to their favorable properties [17,18]. In the literature, no single standardized requirement for the material properties of electrodes dedicated to resistance spot welding of galvanized steels has been established; however, in practice, it is generally assumed that they should exhibit an electrical conductivity of about 40 MS/m (80% IACS), hardness in the range of 140–170 HV, and thermal stability of 450–500 °C [19]. The primary material used for electrode caps is the Cu–Cr–Zr alloy (chromium–zirconium copper), which belongs to the group of precipitation-hardened materials. This alloy, typically containing 0.5–1.2 wt.% Cr and 0.03–0.3 wt.% Zr, is characterized by excellent mechanical properties, with hardness ranging from 70 to 160 HV, high electrical conductivity of up to 75% IACS, and good formability. The CuCr1Zr grade is the most widely applied material for electrode caps in RSW processes, with softening resistance up to 500 °C [20,21,22].

In publications [6,23,24,25], studies were presented concerning Cu–Cr–Zr alloys aimed to improve thermal resistance and electrical conductivity, methods of manufacturing electrode caps, the application of nanolayers on electrode working surfaces, and modifications of electrode geometry to extend electrode life in the resistance spot welding (RSW) of galvanized steels. The Cu–Cr–Zr alloy is characterized by favorable electrical and mechanical properties as well as good plastic deformability, making it suitable as an electrode caps material for welding applications [26,27,28,29,30,31]. Numerous scientific works address modifications of the Cu–Cr–Zr alloy composition to enhance its mechanical and electrical properties. Studies reported in the literature [32,33] indicate that alloying additions to copper and copper alloys influence mechanical property improvements in different ways while typically reducing the electrical conductivity of the alloy. In publication [34], the authors studied Si addition of 0.05 wt.% to the CuCr1.1Zr0.2 alloy, which increased hardness from 207 HV to 229 HV while reducing electrical conductivity from 76.3% IACS to 71.6% IACS after heat treatment. In another study [35], a Fe addition of 0.03 wt.% to CuCr1Zr0.2 resulted in a hardness of 180 HV and 82% IACS after aging at 450 °C for 4 h. The authors of [36] examined the effect of Mg addition (0.05 wt.%) to CuCr0.6Zr0.15; after 40% cold rolling reduction and aging at 500 °C for 0.5 h, the alloy achieved a hardness of 215 HV and an electrical conductivity of 61% IACS. In publication [37], the authors produced a CuCr0.5Zr0.04 alloy with Ti addition of 0.03 wt.%; after hot rolling at 500 °C with 50% reduction and subsequent aging at 500 °C, the material reached an UTS of 350 MPa, corresponding to about 110 HV. The literature also reports studies on rare-earth element additions to Cu–Cr–Zr alloys. In publications [38,39], the effect of Ce addition (0.05 wt.%) to CuCr0.4Zr0.15 was investigated in terms of plastic and service properties. The results showed that after 80% cold rolling reduction and aging at 400 °C for 6 h, the alloy reached a hardness of 183 HV and an electrical conductivity of 83% IACS.

In this article, the results of research on the addition of Sc in amounts of 0.01 wt.% and 0.05 wt.% to Cu–Cr–Zr and Cu–Cr–Ni–Zr alloys are presented, with the aim of increasing hardness while minimizing the decrease in electrical conductivity, and enabling the application of these alloys as materials for electrode caps for the resistance spot welding (RSW) of galvanized steels, with an extended electrode service life. According to the literature review, the addition of Sc in the range of 0.1 wt.% to 0.4 wt.% to copper, depending on the degree of strengthening, results in an increase in hardness up to 214 HV10 and an increase in electrical conductivity up to 44 MS/m when heat treatment is applied. Unfortunately, there is no information available regarding the direct influence of Sc on the Cu-Cr-Zr alloy [26,33,40,41]. Previous studies on the modification of Cu–Cr–Zr alloys [42] have demonstrated that scandium additions to copper and Cu–Cr–Zr alloys positively increase hardness and effectively reduce the diffusion layer in the electrode material during the RSW of galvanized steels. On this basis, work was undertaken to investigate the influence of Sc additions to Cu–Cr–Zr alloys in order to develop a new electrode cap material resistant to wear under RSW conditions for galvanized steel sheets.

## 2. Experimental Study

### 2.1. Materials and Methods

To carry out electrode life tests for the newly developed materials, copper alloys with nominal compositions CuCr0.7Zr0.05 and CuCr0.3Ni0.1Zr0.05 were produced as reference materials, with scandium added in amounts of 0.01 wt.% and 0.05 wt.%. The alloys were synthesized by melting high-purity copper granulate (Cu-ETP) together with alloying additions in the form of pure elements (>99.9%) such as Ni and Sc, or master alloys CuCr7 and CuZr10. Continuous casting on a TERMETAL line (TERMETAL, Piekary Śląskie, Poland) was used to produce rods with a diameter of ø14 mm. All the obtained castings were subjected to chemical composition analysis using a reference standard method and spark-optical emission spectrometry Spectro Spectrotest TX03 (SPECTRO, Kleve, Germany).

In the next stage, the materials were subjected to preliminary heat treatment tests by solution heat treatment at 950 °C and aging at 480 °C (up to 5 h) in a resistance furnace (Czylok, Jastrzębie Zdrój, Poland) in the cold-rolled condition with the true strain εt = 1, corresponding to the average strain of F0-type electrode caps with a diameter of ø16 mm [43,44]. The alloys were then subjected to basic physical property tests after deformation and aging, including Vickers hardness measurements using a Tukon2500 tester (Wilson, Rolling Meadows, IL, USA) and electrical conductivity measurements with a SigmaTest 2.069 device (Foerster, Pittsburgh, PA, USA).

Based on the results of these physical property tests (hardness and electrical conductivity), rods of CuCr0.7Zr0.05 and CuCr0.3Ni0.1Zr0.05Sc0.05 were further processed by cold forging on a hydraulic press (BTC Maszyny, Złobnica, Poland) at KUCA Sp. z o.o. into F0-type electrode caps (ø16 mm × 20 mm) in accordance with ISO 5182:2009 [43].

### 2.2. Electrode Life Tests

**Figure 2:**
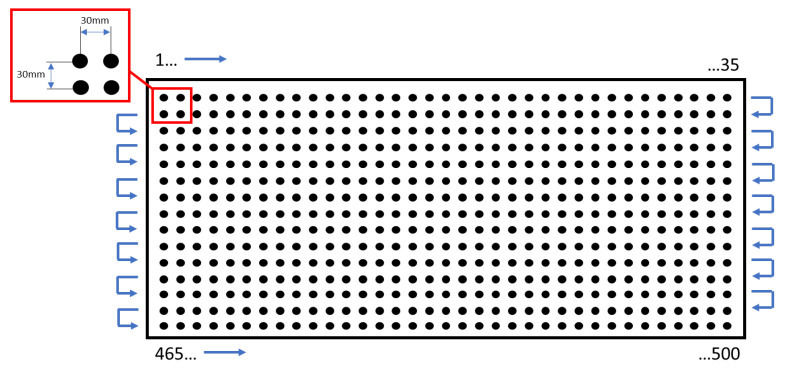
Schematic of the resistance spot welding process of DX53D steel sheet coupons during electrode caps testing.

Electrode life tests of electrode caps made of CuCr0.7Zr0.05, CuCr0.3Ni0.1Zr0.05Sc0.05, and commercial Cu–Cr–Zr electrodes (used as the reference material) in the resistance spot welding (RSW) process were carried out using an inverter-type welding machine under the welding parameters listed in Table 1. The RSW tests were conducted on an inverter-type spot welder manufactured by SMOLTECH (Mokronos Dolny, Poland). The welded material was galvanized steel sheet grade DX53D with a thickness of 0.75 mm, which is commonly applied in the automotive industry for the production of car bodies. The welding tests were performed on sheet coupons according to the configuration shown in Figure 2.

### 2.3. Structural and Mechanical Characterization

**Figure 3:**
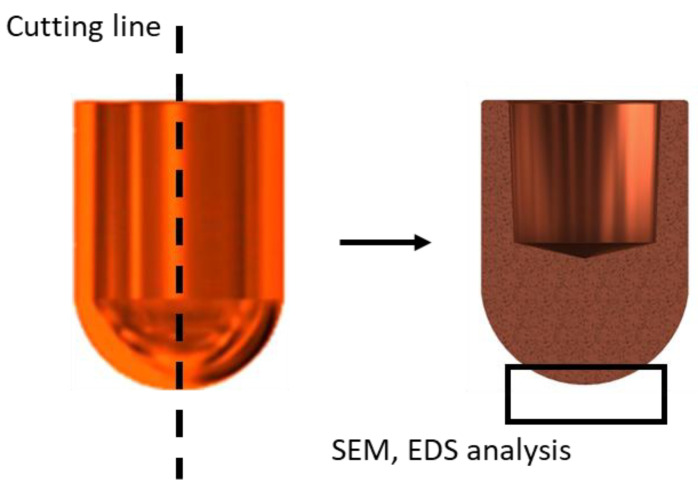
Schematic of microstructural examinations (SEMs) and chemical composition analysis (EDS).

After the resistance spot welding process, the electrode caps were subjected to microstructural examinations in the area of the working face, as shown in Figure 3. SEM imaging (Hitachi SU70, Hitachi, Krefeld, Germany) was used to document the electrode face microstructure, and EDS line scans were used to quantify the thickness of the zinc-rich layer. Thickness was assessed at three separate locations.

The produced spot welds were verified after 250 and 500 joints by performing shear–tension tests using a Zwick/Roell Z100 universal testing machine (Zwick/Roell, Ulm, Germany). The minimum shear–tension strength for spot welds of steel sheets was determined using the following Equation (1) [17,45]:

(1) $$STS=\frac{\left(-6.36\cdot 10^{-7}\cdot R_{m}^{2}+6.58\cdot 10^{-4}\cdot R_{m}+1674\right)\cdot R_{m}\cdot 4\cdot t^{1,5}}{1000}\left(kN\right)$$

where R_m_ is the ultimate tensile strength of the steel (MPa) and t is the sheet thickness (mm). For the DX53D galvanized steel selected for the study, with UTS = 270 MPa, the minimum shear–tension strength for spot welds was determined to be 3580 N.

After the welding tests, the electrode caps underwent 3D geometrical assessment to quantify the mechanical wear of the working face. For this purpose, each tested electrode was 3D-scanned using an ATOS Compact Scan device (Zeiss, Oberkochen, Germany) both before welding and after 500 welds. The study evaluated material loss at the electrode face caused by the diffusion layer in the electrode material and by brass formation, which, under mechanical and electrical loading, is responsible for electrode mushrooming and deterioration of welding conditions.

## 3. Results and Discussion

Within this section, the results of the chemical composition analysis of the individual fabricated copper alloys are first presented, as shown in Table 2.

The results of the chemical composition analysis of the copper alloys indicated that the fabricated electrode caps contained the appropriate amounts of alloying elements, which allowed further experimental investigations to be carried out.

**Figure 4:**
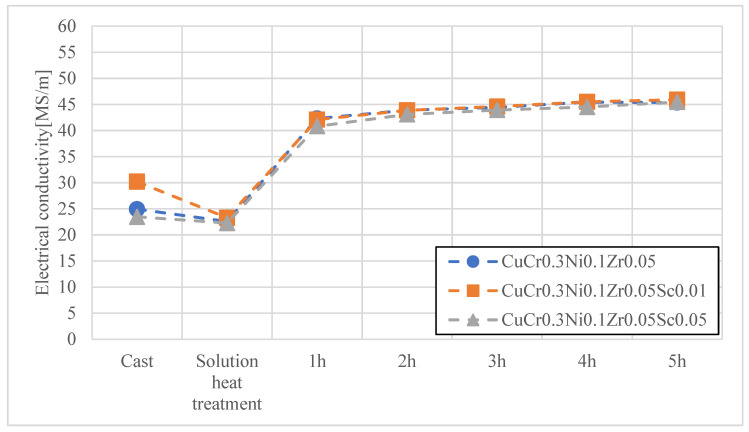
Characteristics of electrical conductivity changes during the aging process of the CuCr0.3Ni0.1Zr0.05 alloy with different Sc contents.

**Figure 5:**
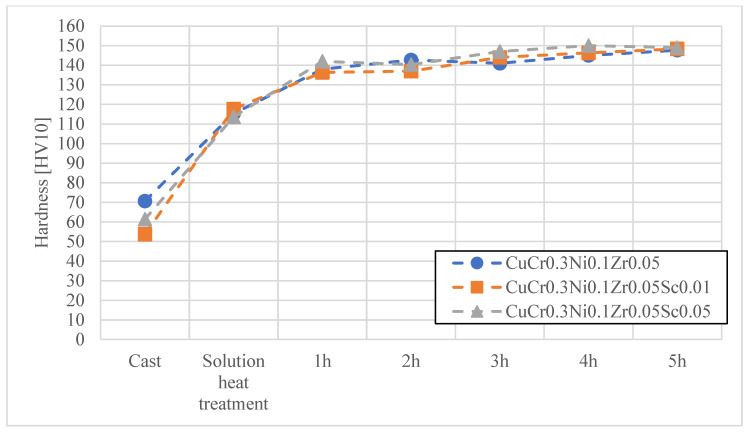
Characteristics of hardness changes during the aging process of the CuCr0.3Ni0.1Zr0.05 alloy with different Sc contents.

**Figure 6:**
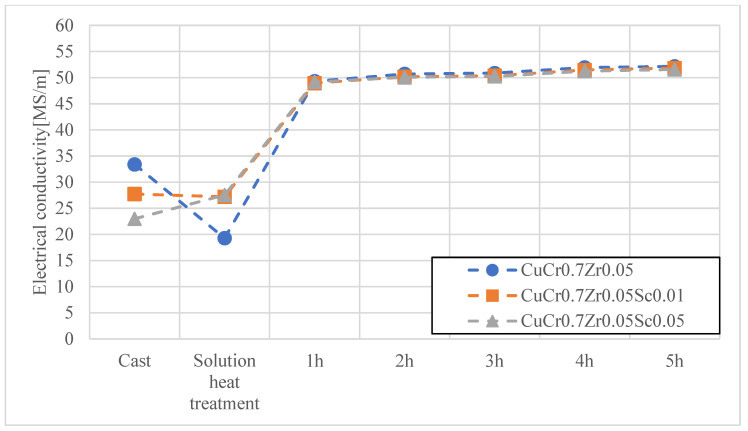
Characteristics of electrical conductivity changes during the aging process of the CuCr0.7Zr0.05 alloy with different Sc contents.

**Figure 7:**
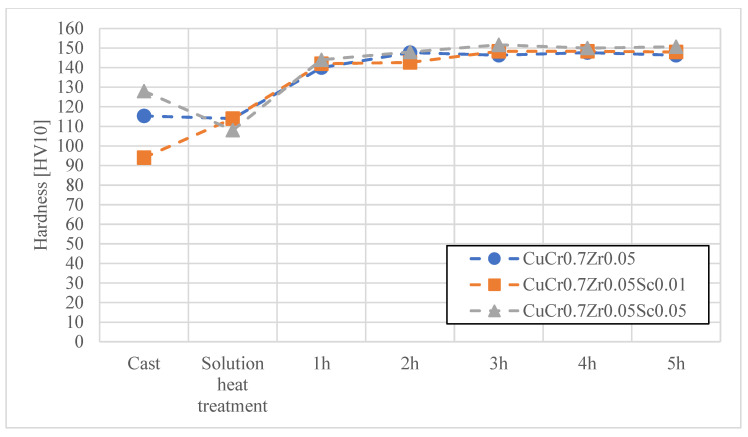
Characteristics of hardness changes during the aging process of the CuCr0.7Zr0.05 alloy with different Sc contents.

In the first stage, heat treatment studies were conducted on the materials developed for electrode caps, namely CuCr0.7Zr0.05 and CuCr0.3Ni0.1Zr0.05 with Sc additions of 0.01 wt.% and 0.05 wt.%. The materials obtained by continuous casting were subjected to cold working, followed by an aging process at 480 °C for up to 2 h. Figure 4, Figure 5, Figure 6 and Figure 7 present the characteristics of the changes in alloy properties in terms of hardness and electrical conductivity during aging.

The conducted studies on the influence of scandium content in the developed copper alloys, i.e., CuCr0.7Zr0.05 and CuCr0.3Ni0.1Zr0.1, showed that scandium additions up to 0.05 wt.% do not significantly affect electrical conductivity, while slightly increasing the hardness of the alloys during the aging process. The most favorable properties in terms of achieving maximum hardness were observed under the following conditions: after 3 h of aging, the CuCr0.7Zr0.05 alloy exhibited a hardness of about 152 HV10 and an electrical conductivity of 51.2 MS/m; after 4 h of aging, the CuCr0.3Ni0.1Zr0.1 alloy showed a hardness of about 150 HV10 and an electrical conductivity of 45.5 MS/m. Due to the minimal effect of Sc on the decrease in electrical conductivity of the studied alloys, CuCr0.7Zr0.05Sc0.05 and CuCr0.3Ni0.1Zr0.1Sc0.05 alloys were selected for electrode cap testing as potentially the most resistant to the diffusion layer from welded galvanized sheets.

**Figure 8:**
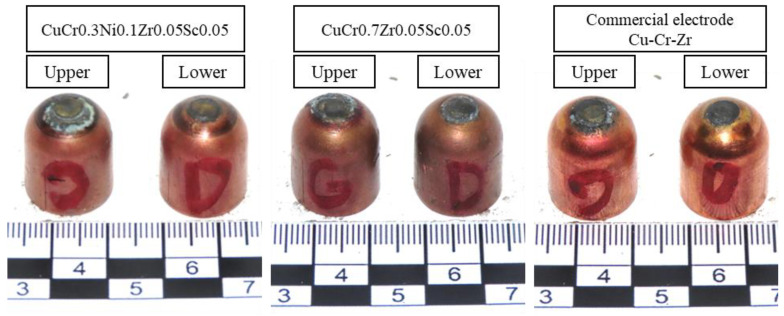
View of electrode cap surfaces after 500 spot welds.

In the next stage of the study, tests were carried out on the F0-type Ø16 electrode caps developed in this work during the resistance spot welding (RSW) of galvanized steel sheets. Figure 8 shows the appearance of the electrodes after the welding process.

Observations made during the resistance spot welding process showed that, for both CuCr0.7Zr0.05Sc0.05 and CuCr0.3Ni0.1Zr0.1Sc0.05 electrodes, no arcing was observed during nugget formation between the joined sheets. In contrast, when analyzing the performance of the commercial electrode, it was found that arcing occurred during the first ~20 spot welds. Furthermore, adhesion of the electrode caps to the sheet surface was observed when performing welds beyond weld number 400, which indicates disturbed welding conditions associated with electrode wear.

**Figure 9:**
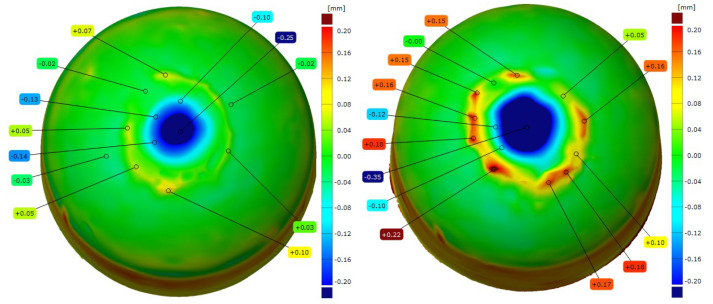
**Three–dimensional** geometric view of CuCr0.3Ni0.1Zr0.1Sc0.05 electrode caps after the welding process (**left**: lower electrode, **right**: upper electrode).

**Figure 10:**
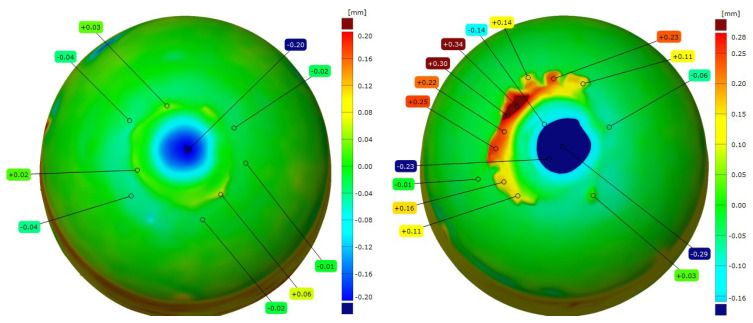
**Three–dimensional** geometric view of CuCr0.7Zr0.05Sc0.05 electrode caps after the welding process (**left**: lower electrode, **right**: upper electrode).

**Figure 11:**
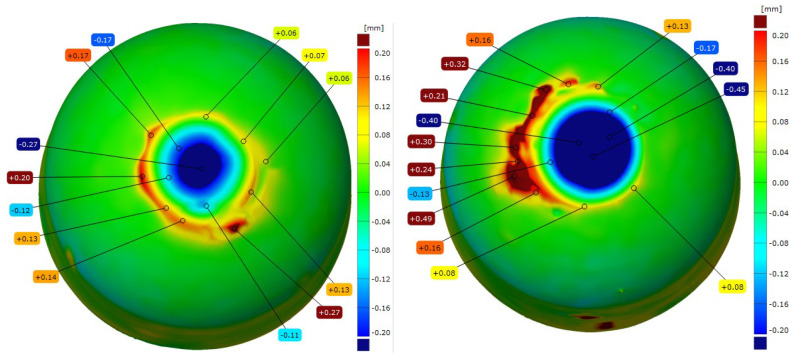
**Three–dimensional** geometric view of commercial Cu–Cr–Zr electrode caps after the welding process (**left**: lower electrode, **right**: upper electrode).

**Figure 12:**
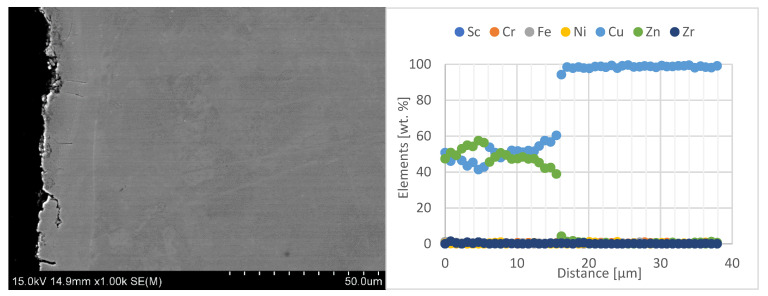
Microstructure of the lower CuCr0.3Zr0.1Ni0.1Sc0.05 electrode after industrial welding tests (1000× magnification) and EDS line-scan chemical composition analysis.

After the resistance spot welding of galvanized steel sheets, the electrode caps were examined for changes in the geometry of the working face using 3D scanning technology. Figure 9, Figure 10, Figure 11 and Figure 12 show the scanned views of the electrode working faces, and Table 3 summarizes the results of electrode height loss after tests.

When analyzing the results obtained for the geometry of the electrode caps after conducting welding tests on the galvanized sheets, it can be concluded that the lower electrodes exhibit less working face wear than the upper electrodes, which directly contact and press the sheets during welding. This phenomenon leads to the gradual mushrooming of the working faces, which, in combination with the diffusion layer and brass formation, causes accelerated electrode wear. The results of the geometry measurements showed that commercial Cu–Cr–Zr electrodes displayed higher wear than the new Sc-modified electrodes by 0.02–0.07 mm (lower electrode) and 0.10–0.16 mm (upper electrode).

**Figure 13:**
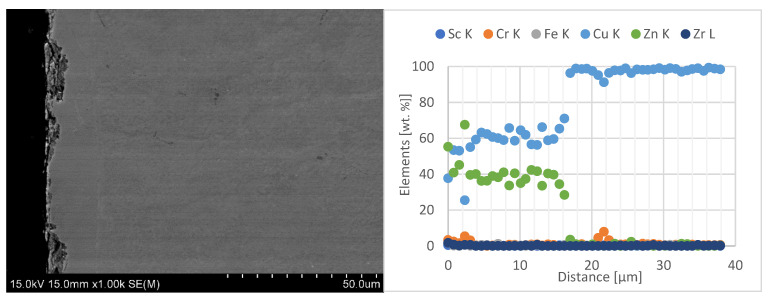
Microstructure of the lower CuCr0.7Zr0.05Sc0.05 electrode after industrial welding tests (1000× magnification) and EDS line-scan chemical composition analysis.

**Figure 14:**
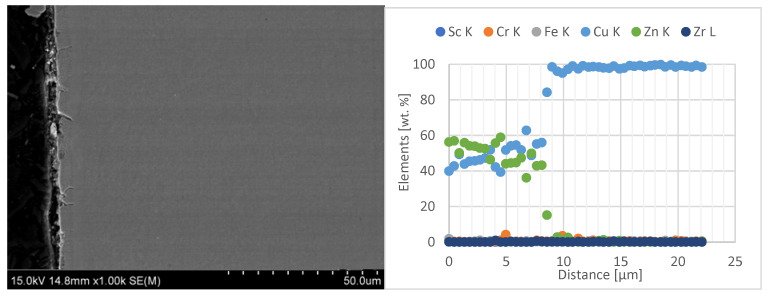
Microstructure of the lower commercial Cu–Cr–Zr electrode after industrial welding tests (1000× magnification) and EDS line-scan chemical composition analysis.

In the next stage of the electrode caps study, the results of zinc penetration measurements in the electrode faces were presented. The tests were carried out using line-scan EDS chemical composition analysis with SEM on the central part of the lower electrode caps, as shown in Figure 12, Figure 13 and Figure 14.

When analyzing the obtained results of the electrode working faces with respect to the diffusion layer, it can be observed that the newly developed Sc-modified electrode caps, compared with commercial Cu–Cr–Zr electrodes, exhibit significantly lower zinc penetration into the working face. Electrode caps forged from CuCr0.3Zr0.1Ni0.1Sc0.05 and CuCr0.7Zr0.05Sc0.05 alloys are characterized by a diffusion-layer zone about half as thick, in the range of 8–12 µm, whereas the commercial Cu–Cr–Zr electrode shows a zinc penetration depth of about 20 µm. A detailed summary of the results is presented in Table 4.

**Figure 15:**
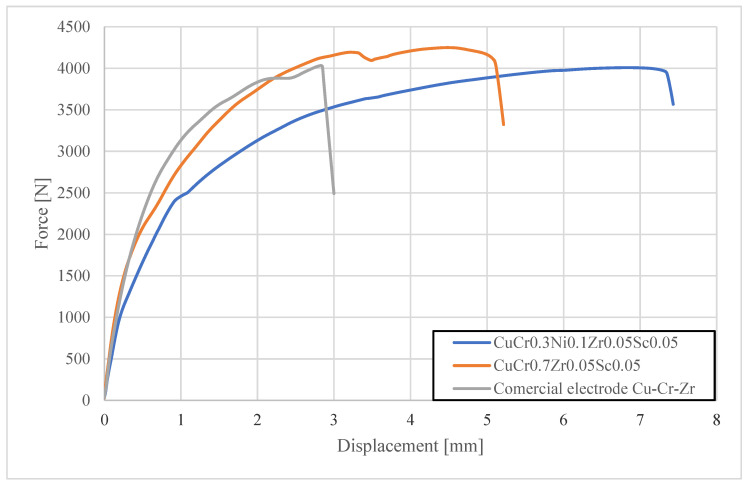
Characteristics of shear strength tests for welds produced using electrode caps (after 250 welds).

**Figure 16:**
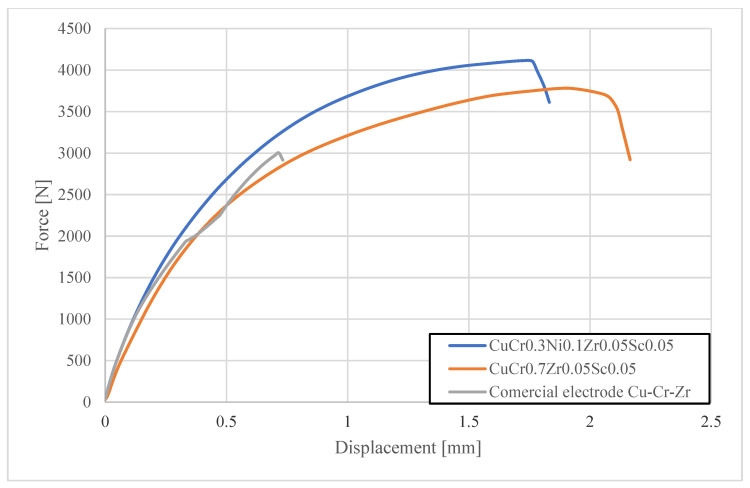
Characteristics of shear strength tests for welds produced using electrode caps (after 500 welds).

In the final test, the fracture force in shear–tension tests was measured for spot welds produced after 250 and 500 welds using different types of electrode caps. The tests were carried out with a Zwick/Roell Z100 universal testing machine. Figure 15 and Figure 16 present the load–elongation curves of the fracture forces.

The shear strength tests showed that spot welds produced with CuCr0.3Zr0.1Ni0.1Sc0.05 and CuCr0.7Zr0.05Sc0.05 electrodes exhibited similar fracture forces of about 4000–4200 N after 250 welds, meeting the requirements for joint quality. However, after 500 welds, the spot welds made with CuCr0.3Ni0.1Zr0.05Sc0.05 electrodes demonstrated a significantly higher fracture force of 4090 N, and those made with CuCr0.7Zr0.05Sc0.05 electrodes reached 3780 N, compared with 2910 N for welds made using commercial CuCr0.7Zr0.05 electrodes. Moreover, the shape of the shear curve for welds produced with the commercial electrodes indicated a lack of nugget fusion in the spot weld nugget.

The results obtained in this study clearly indicate the beneficial effect of scandium additions to Cu–Cr–Zr and Cu–Cr–Ni–Zr alloys intended for electrode caps in resistance spot welding (RSW) of galvanized steels. The noticeable increase in hardness with only a minimal reduction in electrical conductivity, along with the suppression of the diffusion layer and reduced mechanical wear of the electrodes, confirms the validity of this direction of material modification. In the context of the available literature, these results are consistent with earlier studies on improving Cu–Cr–Zr alloys through alloying additions. For example, Zhang et al. [38,39] demonstrated that cerium (Ce) additions also improve hardness and electrical conductivity in Cu–Cr–Zr alloys, but scandium appears more advantageous due to its simultaneous effect of limiting the diffusion layer in the electrode structure, which represents a critical issue when welding zinc-coated components. The presented results show that the diffusion layer zone for Sc-modified electrodes does not exceed 12.4 µm, which is about 40% lower compared to conventional Cu–Cr–Zr electrodes. A similar phenomenon of reducing electrode degradation by material modifications was also reported by Zou and Zhao [16], who applied surface modifications to increase electrode durability, although this required additional production steps. The solution proposed in this article, based on bulk chemical modification of the electrode material, achieves a comparable effect while maintaining manufacturing simplicity. In terms of mechanical resistance, it was observed that Sc-modified electrodes, both CuCr0.7Zr0.05Sc0.05 and CuCr0.3Ni0.1Zr0.05Sc0.05, showed significantly lower geometric wear of the working face compared to the reference Cu–Cr–Zr electrodes. These results are consistent with the findings of Mahmud et al. [14], who demonstrated that cap-face degradation is closely linked to diffusion layer and brass layer formation in the electrode–sheet contact zone, ultimately leading to accelerated wear. In this context, scandium, by limiting zinc penetration, has a positive effect on electrode durability. Equally important is the quality of the spot welds themselves. Shear–tension tests showed that after 500 welds, joints produced with Sc-modified electrodes exceeded both the minimum normative requirements (3580 N) and the values obtained with commercial electrodes (2910 N), reaching 4090 N for CuCr0.3Ni0.1Zr0.05Sc0.05. These findings correlate with the observations of Vabai et al. [45], who reported a significant reduction in spot weld quality as electrodes wear, emphasizing the importance of improving electrode material properties to maintain high production quality. It is also noteworthy that the present research aligns with current global trends in the search for new RSW electrode materials, as evidenced by works such as Ping et al. [23], who investigated TiB_2_ nanolayers on electrode surfaces, and Liu et al. [36], who analyzed the effect of Mg additions on Cu–Cr–Zr alloys. However, the approach proposed in this paper, based on scandium additions to Cu–Cr–Ni–Zr and Cu–Cr–Zr alloys, offers a more comprehensive solution, eliminating the need for additional technological operations such as coating or laser hardening.

## 4. Conclusions

The experimental studies on the fabrication and properties of new Cu–Cr–Ni–Zr and Cu–Cr–Zr alloys with Sc additions have led to the following statements and conclusions:

1. The conducted research clearly confirmed that the modification of Cu–Cr–Zr and Cu–Cr–Ni–Zr alloys through controlled scandium (Sc) additions contributes to a significant improvement in the service properties of electrode caps used in the resistance spot welding (RSW) of galvanized steels. The applied alloying concept allows for increased electrode hardness with only a minimal reduction in electrical conductivity, remaining within acceptable limits for this type of application.
2. Moreover, the developed alloy compositions effectively reduce the diffusion layer in the electrode material, which is a key factor leading to the degradation of the electrode face geometry and to accelerated wear during welding. Compared with commercial Cu–Cr–Zr electrodes, the newly developed Sc-modified electrodes exhibited about 40% reduction in the diffusion-layer-zone thickness and a distinct decrease in material loss from the electrode working surface, as confirmed by 3D geometry analysis.
3. Mechanical tests of the produced spot welds showed that the use of Sc-modified electrodes ensures high shear strength of welds, exceeding both the minimum normative requirements and the performance of joints made with commercial electrode caps after 500 welds.
4. The results obtained are consistent with current global research trends focused on increasing electrode durability through microstructural and compositional modifications. However, it should be emphasized that the proposed solution, based on the modification of the chemical composition of the electrode material, offers a technological advantage over surface engineering methods by simplifying the manufacturing process and eliminating the need for additional technological treatments.

To fully verify the potential of the developed materials, further investigations under industrial conditions are recommended, involving a significantly higher number of welds and the influence of typical factors present in production environments.

## Figures and Tables

**Table 1:** Process parameters for electrode caps testing.

Parameter	Value
Number of spot welds	500
Number of current pulses	2
Initial welding current	6500 A
Welding current increase	500 every 100 welds
Thickness of welded materials	0.75 mm
Type of welded materials	DX53D
UTS of welded materials	270 MPa

**Table 2:** Summary of the fabricated copper alloy materials with their chemical composition analysis.

No.	Material	Elements [wt. %]					
		Cr	Zr	Ni	Sc	Others	Cu (Rest)
1	CuCr0.7Zr0.05	0.68	0.06	-	-	<0.01	99.25
**2**	CuCr0.7Zr0.05Sc0.01	0.67	0.07	-	0.01	<0.01	99.24
**3**	CuCr0.7Zr0.05Sc0.05	0.68	0.06	-	0.06	<0.01	99.19
4	CuCr0.3Ni0.1Zr0.05	0.31	0.05	0.11	-	<0.01	99.52
**5**	CuCr0.3Ni0.1Zr0.05Sc0.01	0.3	0.06	0.11	0.01	<0.01	99.51
**6**	CuCr0.3Ni0.1Zr0.05Sc0.05	0.31	0.05	0.11	0.05	<0.01	99.47

**Table 3:** Summary of working face material loss of cap electrodes after welding tests.

Type of Electrode	Lower Electrode [mm]	Upper Electrode [mm]
CuCr0.3Zr0.1Ni0.1Sc0.05	−0.25	−0.35
CuCr0.7Zr0.05Sc0.05	−0.2	−0.29
Commercial electrode Cu-Cr-Zr	−0.27	−0.45

**Table 4:** Summary of diffusion layer test results for the lower electrode after 500 resistance spot welds.

Type of Electrode	Average Value of Diffusion Layer [µm]	SD
CuCr0.3Zr0.1Ni0.1Sc0.05	8.28	0.8
CuCr0.7Zr0.05Sc0.05	12.4	1.05
Commercial electrode Cu-Cr-Zr	19.9	0.75

## Data Availability

The original contributions presented in this study are included in the article. Further inquiries can be directed to the corresponding author.

## References

[1] (2025). Economic and Market Report, Global and EU Auto Industry: Full Year 2024, ACEA. https://www.acea.auto/files/Economic_and_Market_Report-Full_year-2024.pdf.

[2] Akkaş N. (2017). Welding Time Effect on Tensile-Shear Loading in Resistance Spot Welding of SPA-H Weathering Steel Sheets Used in Railway Vehicles. Acta Phys. Pol. A.

[3] Rdzawski Z., Kwaśniewski P., Głuchowski W., Łagoda M., Maleta M., Boczkal S., Franczak K. (2023). Research on changes in microstructures and mechanical properties of Welding caps as a result of their usage during resistance spot welding process. Arch. Met. Mater..

[4] Choudhari R., Adhaye A., Sulakhe V., Fegade R. (2025). Recent Status of Research and Developments in Resistance Spot Welding. Int. J. Mech. Eng..

[5] Başkaya Ü., Uzun R., Atapek Ş.H., Kılıç Y., Polat Ş. (2024). Effect of coating type on electrode degradation and its life in resistance spot welding of a low carbon steel. Eng. Fail. Anal..

[6] Mazur W., Kyriakopoulos A., Bott N., West D. (2016). Use of modified electrode caps for Surface quality welds in resistance spot welding. J. Manuf. Process..

[7] Lin H., Hsu C., Lee C., Kuo T., Jeng S. (2018). Effects of zinc layer thickness on Resistance spot Welding of galvanized mild steel. J. Mech. Work. Technol..

[8] Sheikhi M., Valaee-Tale M., Mazaheri Y., Usefifar G.R. (2024). Electrode lifetime in resistance spot welding of coated sheets: Experiments and modeling. Mater. Today Commun..

[9] Mikno Z., Bartnik Z. (2016). Heating of electrodes during spot Resistance Welding in FEM calculations. Arch. Civ. Mech. Eng..

[10] Song S., Shojaee M., Midawi A., Sherepenko O., Ghassemi-Armaki H., Biro E. (2023). Influence of expulsion and heat extraction resulting from changes to electrode force on liquid metal embrittlement during resistance spot welding. J. Mater. Res. Technol..

[11] Blondeau R. (2008). Metallurgy and Mechanics of Welding—Processes and Industrail Applications.

[12] Panza L., De Maddis M., Spena P.R. (2022). Use of electrode displacement signals for electrode degradation assessment in Resistance spot Welding. J. Manuf. Process..

[13] Ghatei-Kalashami A., Zhang S., Shojaee M., Midawi A.R.H., Goodwin F., Zhou N.Y. (2022). Failure behawior of Resistance spot welded advanced high strength steel: The role of Surface condition and initial microstructure. J. Mech. Work. Technol..

[14] Mahmud K., Murugan S.P., Cho Y., Ji C., Nam D., Park Y.-D. (2021). Geometrical degradation of electrode and liquid metal embrittlement cracking in resistance spot welding. J. Manuf. Process..

[15] Zhang W., Sun D., Han L., Li Y. (2015). Optimised design of electrode morphology for novel dissimilar Resistance spot Welding of aluminium alloy and galvanised high strength steel. Mater. Des..

[16] Zou J., Zhao Q., Chen Z. (2009). Surface modified long-life electrode for Resistance spot Welding of Zn-coated steel. J. Mech. Work. Technol..

[17] (2019). AWS Recommended Practices for Resistance Welding.

[18] (2016). Resistance Welding—Materials for Electrodes and Ancillary Equipment.

[19] Zhang H., Senkara J. (2012). Resistance Welding.

[20] Tu J.P., Qi W.X., Yang Y.Z., Liu F., Zhang J.T., Gan G.Y., Wang N.Y., Zhang X.B., Liu M.S. (2002). Effect of aging treatment on the electrical sliding wear behawior of Cu-Cr-Zr alloy. Wear.

[21] Sarin V.K., Grant N.J. (1972). Cu-Zr and Cu-Zr-Cr alloys produced from rapidly quenched powders. Met. Trans..

[22] Batra I.S., Dey G.K., Kulkarni U.D., Banerjee S. (2001). Microstructure and properties of a Cu-Cr-Zr alloy. J. Nucl. Mater..

[23] Ping L., Shijie D., Zhixiong X., Anzhuo Y., Wei Y. (2014). The effects of coating parameters on the quality of TiB2–TiC composite phase coating on the surface of Cu–Cr–Zr alloy electrode. Surf. Coat. Technol..

[24] Dong S.J., Zhou Y. (2003). Effects of TiC composite coating on electrode degradation in microresistance welding of nickel-plated steel. Met. Mater. Trans. A.

[25] Cheng L., Shijie D., Xiang X., Norman Z. (2009). Mass loss of copper alloy electrode during TiB2 coating by electrospark deposition. Surf. Coat. Technol..

[26] Franczak K., Kwaśniewski P., Kiesiewicz G., Zasadzinska M., Jurkiewicz B., Strzepek P., Rdzawski Z. (2020). Research of mechanical and electrical properties of Cu-Sc and Cu-Zr alloys. Arch. Civ. Mech. Eng..

[27] Lin G.B., Wang Z.D., Zhang M.K., Zhang H., Zhao M. (2011). Heat treatment method for making high strength and conductivity Cu–Cr–Zr alloy. Mater. Sci. Technol..

[28] Mishnev R., Shakhova I., Belyakov A., Kaibyshev R. (2015). Deformation microstructures, strengthening mechanisms, and electrical conductivity in a Cu–Cr–Zr alloy. Mater. Sci. Eng. A.

[29] Lipinska M., Bazarnik P., Lewandowska M. (2014). The electrical conductivity of CuCrZr alloy after SPD processing. IOP Conference Series Materials Science and Engineering.

[30] Yan F., Chen W., Feng P., Dong L., Yang T., Ren S., Fu Y. (2020). Microstructure evolution and enhanced properties of Cu–Cr–Zr alloys through synergistic effects of alloying, heat treatment and low-energy cyclic impact. J. Mater. Res..

[31] Ostachowski P., Bochniak W., Łagoda M., Ziółkiewicz S. (2019). Strength properties and structure of CuCrZr alloy subjected to low-temperature KOBO extrusion and heat treatment. Int. J. Adv. Manuf. Technol..

[32] Davis J.R. (2001). ASM Specialty Handbook: Copper and Copper Alloys.

[33] Franczak K., Sadzikowski M., Kwaśniewski P., Kiesiewicz G., Ściężor W., Kordaszewski S. (2024). Research on Alloying Elements’ Influence on CuETP-Grade Copper’s Mechanical and Electrical Properties. Materials.

[34] Wang W., Zhang Y., Yang H., Su L., Wang C., Tong C., Zhou J., Chen J., Wang B. (2022). Effects of Si addition on properties and microstructure of CuCrZr alloy. J. Alloys Compd..

[35] Zhou H.T., Zhong J.W., Zhou X., Zhao Z.K., Li Q.B. (2008). Microstructure and properties of Cu–1.0Cr–0.2Zr–0.03Fe alloy. Mater. Sci. Eng. A.

[36] Liu P., Kang B.X., Cao X.G., Huang J.L., Yen B., Gu H.C. (1999). Aging precipitation and recrystallization of rapidly solidified Cu–Cr–Zr–Mg alloy. Mater. Sci. Eng. A.

[37] Chenna Krishna S., Sudarsana Rao G., Jha A.K., Pant B., Venkitakrishnan P.V. (2016). Strengthening in high strength Cu-Cr-Zr-Ti alloy plates produced by hot rolling. Mater. Sci. Eng. A.

[38] Zhang Y., Volinsky A.A., Tran H.T., Chai Z., Liu P., Tian B., Liu Y. (2016). Aging behavior and precipitates analysis of the Cu–Cr–Zr–Ce alloy. Mater. Sci. Eng. A.

[39] Zhang Y., Volinsky A.A., Tran H.T., Chai Z., Liu P., Tian B. (2015). Effects of Ce Addition on High Temperature Deformation Behavior of Cu-Cr-Zr Alloys. J. Mater. Eng. Perform..

[40] Franczak K. (2025). Influence of heat treatment and plastic deformation on the mechanical and electrical properties of Cu-Sc alloys prepared by continuous casting process. Arch. Foundry Eng..

[41] Franczak K., Kwaśniewski P., Kiesiewicz G., Sadzikowski M., Sciezor W., Kordaszewski S., Kuca D. (2022). Research on the continuous casting process of CuCrZr alloys with the addition of scandium dedicated for Resistance Welding electrodes. Metalurgija.

[42] Franczak K., Kwaśniewski P., Kiesiewicz G., Ściężor W., Sadzikowski M., Kordaszewski S., Noga P. (2025). Research on zinc diffusion in the resistance spot welding process of galvanised steels using Cu-Sc and Cu-Zr alloy electrodes. Weld. World.

[43] (2009). Spot Welding Electrode Caps.

[44] Franczak K. (2023). Research on Cu-Sc Alloys Dedicated for Resistance Welding Electrodes. Ph.D. Thesis.

[45] Vabai B., Sommer C., Szabo M., Toth T., Majlinger K. (2019). Shear tension strength of resistance spot welded ultra high strength steel. Thin-Walled Struct..

